# Human Neutrophil Granule Exocytosis in Response to *Mycobacterium smegmatis*

**DOI:** 10.3390/pathogens9020123

**Published:** 2020-02-15

**Authors:** Irina Miralda, Christopher K. Klaes, James E. Graham, Silvia M. Uriarte

**Affiliations:** 1 Department of Microbiology & Immunology, School of Medicine, University of Louisville, 505 S. Hancock St., Louisville, KY 40202, USA; irina.miralda@louisville.edu; 2 Department of Medicine, School of Medicine, University of Louisville, 570 S. Preston St., Louisville, KY 40202, USA; keith.klaes@louisville.edu

**Keywords:** human neutrophils, granule exocytosis, environmental mycobacteria

## Abstract

*Mycobacterium smegmatis* rarely causes disease in the immunocompetent, but reported cases of soft tissue infection describe abscess formation requiring surgical debridement for resolution. Neutrophils are the first innate immune cells to accumulate at sites of bacterial infection, where reactive oxygen species and proteolytic enzymes are used to kill microbial invaders. As these phagocytic cells play central roles in protection from most bacteria, we assessed human neutrophil phagocytosis and granule exocytosis in response to serum opsonized or non-opsonized *M. smegmatis* mc^2^. Although phagocytosis was enhanced by serum opsonization, *M. smegmatis* did not induce exocytosis of secretory vesicles or azurophilic granules at any time point tested, with or without serum opsonization. At early time points, opsonized *M. smegmatis* induced significant gelatinase granule exocytosis compared to non-opsonized bacteria. Differences in granule release between opsonized and non-opsonized *M. smegmatis* decreased in magnitude over the time course examined, with bacteria also evoking specific granule exocytosis by six hours after addition to cultured primary single-donor human neutrophils. Supernatants from neutrophils challenged with opsonized *M. smegmatis* were able to digest gelatin, suggesting that complement and gelatinase granule exocytosis can contribute to neutrophil-mediated tissue damage seen in these rare soft tissue infections.

## 1. Introduction

*Mycobacterium smegmatis* is a rod-shaped, Gram-positive, environmental mycobacterium that typically inhabits soils and natural and municipal waters, leading to frequent human exposure [1]. While fewer than 100 cases of infection have been reported, *M. smegmatis* can cause post-traumatic or post-operative soft tissue infections that remain localized unless the patient is immunocompromised [2,3,4,5,6,7,8,9,10,11,12,13,14]. Diagnosis, treatment, and management of these and other similarly rare environmental mycobacterial infections poses a challenge to medical providers. *M. smegmatis* typically causes a recurrent infection where antibiotic courses are ineffective. This results in a general histopathology of chronic skin ulceration with violaceous edges, rolled margins, and significant subcutaneous necrosis that requires aggressive debridement of all infected tissues and skin [12]. *M. smegmatis* and other saprophytic mycobacteria share a unique, lipid-rich, hydrophobic cell wall architecture that confers a high tolerance to physical and chemical damage and contributes to protection against host oxidative and other defense mechanisms [15,16,17]. Components from the mycobacterial cell wall also have immunomodulatory capabilities that contribute to their virulence. For example, *M. smegmatis* expresses a cell surface phosphoinositol-capped lipoarabinomannan (PILAM) that has previously been shown to contribute to neutrophil granule mobilization, and is capable of priming the release of extracellular gelatinase [18].

Individuals with low peripheral neutrophil counts dramatically demonstrate the central role of neutrophils in human host immunity to bacterial infection. These patients are susceptible to a wide range of bacterial infections with increased frequency, severity, and overall mortality [19]. Both oxidation-dependent and independent neutrophil killing mechanisms contribute to protection against microbial invaders [20,21]. Oxygen-independent killing involves vesicular trafficking of gelatinase, specific and azurophilic granules. These are targeted internally towards a bacteria-containing phagosome, or to the plasma membrane, targeting extracellular bacteria. The four granule subtypes differ from each other in terms of their protein content, density, and a hierarchy in terms of toxicity and stimulus intensity required for their mobilization [22,23]. As granule content becomes more potentially destructive, degranulation becomes more tightly regulated, requiring stronger stimuli to evoke exocytosis [22,23]. Despite their beneficial microbicidal roles, the uncontrolled or prolonged release of these granules results in damage and pathologic changes to host tissues.

Recognition of both slow-growing and environmental mycobacteria by phagocytic cells has been shown to involve human mannose-binding lectin, which allows activation of the complement lectin pathway and increase phagocytosis [24]. A prior study of the specific interactions between human neutrophils and *M. smegmatis* (using strain ATCC 607) showed that both live and antibody opsonized dead bacteria were both able to inhibit azurophilic granule–phagosome fusion, and exocytosis of these vesicles [25]. Live bacteria were previously shown by the same researchers to evoke the extracellular release of both specific granules, and reactive oxygen species [26]. Both of those studies were performed using a high multiplicity of infection (50 bacteria per phagocyte), where other studies of mycobacteria have indicated that both bacterial clumping and levels can evoke different immune cell responses. A limited number of additional studies that have examined *M. smegmatis* interactions with human neutrophil [27,28,29,30,31] have focused on the intracellular killing of the bacterium, but not on neutrophil granule release. Therefore, we sought to characterize the granule release pattern of human neutrophils evoked by *M. smegmatis* in vitro and determine whether serum opsonization has any influence on this interaction. We found that opsonization facilitated phagocytosis of *M. smegmatis*, but regardless of opsonization, neither secretory vesicles, nor azurophilic granules were released in our assays. At early time points, only opsonized *M. smegmatis* triggered the release of gelatinase granules capable of degrading extracellular matrix proteins. At later time points, both non-opsonized and serum opsonized *M. smegmatis* induced significant gelatinase granule and specific granule exocytosis. Our results suggest that neutrophils and complement have a direct contribution to the tissue destruction observed in the cases of *M. smegmatis* infection. 

## 2. Results

### 2.1. Opsonization Increases Neutrophil-M. smegmatis Interactions 

**Figure 1:**
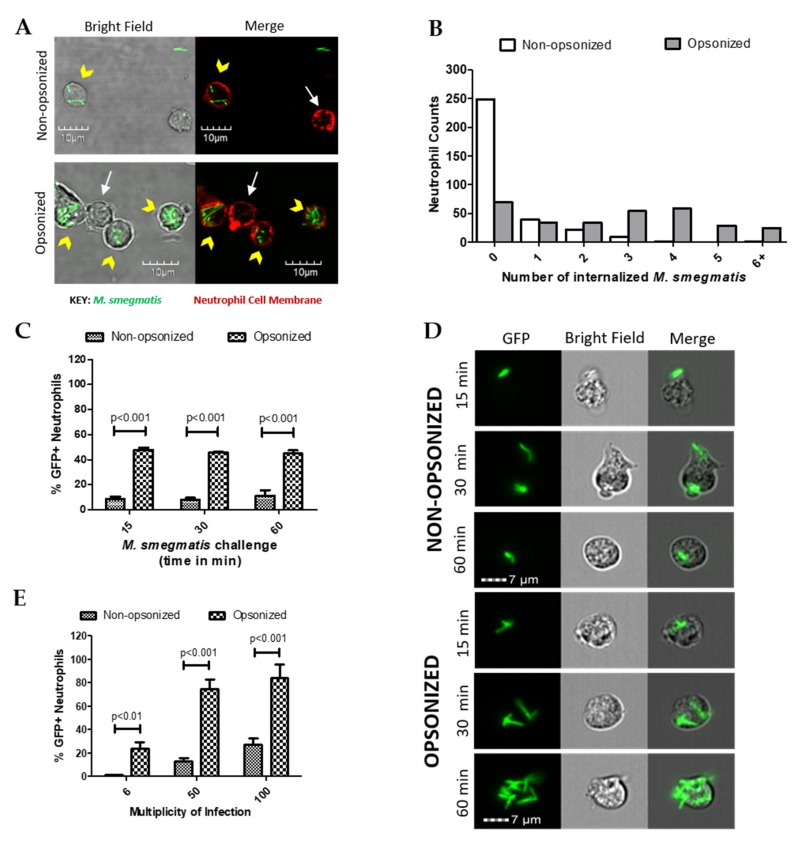
The effect of opsonization on phagocytosis of *M. smegmatis* by neutrophils was observed via confocal microscopy (**A**). Neutrophil cell membranes were stained with Wheat Germ Agglutinin (WGA, red) and GFP-expressing *M. smegmatis* was utilized. Yellow arrowheads show neutrophils that phagocytized *M. smegmatis* and white arrows point to uninfected neutrophils. The number of bacteria was counted in 100 neutrophils after 60 min of co-culture with either opsonized or non-opsonized *M. smegmatis* (**B**). Data are expressed as the number of neutrophils per number of internalized bacteria from three experiments. Internalization was also measured by Imagestream analysis where 2000 neutrophil events were collected and quantified based on GFP expression after either 15, 30, and 60 min of challenge with *M. smegmatis* at a multiplicity of infection (MOI) of 6 (**C**) or after 60 min at a MOI of 6, 50 or 100 (**E**). Representative images from the time course experiment are shown (**D**). Data for both Imagestream experiments are expressed as the mean ± SEM of the average %GFP neutrophils from three experiments. Two-way ANOVAs followed by Bonferroni post-hoc tests were used to determine significance.

The role of opsonization in neutrophil uptake of green fluorescent protein (GFP)-expressing *M. smegmatis* was initially assessed using confocal microscopy. Following co-culture, neutrophil cell membranes were fluorescently labeled to visualize internalized and attached *M. smegmatis* (Figure 1A). Neutrophils were counted and classified based on whether they had any bacteria associated; those with *M. smegmatis* associated were further classified based on the location of their associated bacterial location (Table 1). As expected, the phagocytic efficiency of neutrophils increased when bacteria were opsonized. The percentage of neutrophils with no associated bacteria decreased from 69% in the non-opsonized condition to 19% when co-cultured with opsonized *M. smegmatis*. Furthermore, opsonization increased the number of neutrophils with attached and internalized bacteria or had both internalized and attached bacteria. Serum opsonization did increase the number of cells that only had bacteria attached. Neutrophils challenged with either non-opsonized or opsonized *M. smegmatis* were classified based on the number of internalized bacteria (Figure 1B). More neutrophils phagocytized a higher number of opsonized bacteria, whereas very few neutrophils internalized non-opsonized *M. smegmatis*. Together, our data show that a larger population of neutrophils are internalizing greater numbers of *M. smegmatis* when it is serum opsonized. 

The internalization kinetics of *M. smegmatis* by human neutrophils were then studied during a time course of 15, 30, or 60 min of co-culture in suspension (Figure 1C). At each time point tested, the population of neutrophils that internalized opsonized *M. smegmatis* was significantly larger than the non-opsonized condition. Notably, similar percentages of GFP positive neutrophils remained from 15 to 60 min of co-culture, which suggests that phagocytosis of *M. smegmatis* largely takes place in the first minutes of interaction regardless of bacterial treatment. Visualization of GFP+ cells by Imagestream confirmed that the number of bacteria internalized increased considerably in the opsonized conditions in a time-dependent manner (Figure 1D). 

Next, the phagocytic capacity of neutrophils was tested in a dose response experiment where they were exposed to GFP-expressing *M. smegmatis* at increasing multiplicities of infection (MOIs). At all MOIs tested, the population of GFP+ cells was significantly larger when neutrophils were co-cultured with opsonized *M. smegmatis* compared to non-opsonized bacteria (Figure 1E). Co-culture of neutrophils with opsonized, GFP-expressing *M. smegmatis* at a MOI of 50 and 100 resulted in nearly 100% of neutrophils becoming GFP positive, indicating a high incidence of *M. smegmatis* phagocytosis. Contrastingly, even at a MOI of 100 with non-opsonized *M. smegmatis*, less than 40% of neutrophils became GFP+, which suggests that *M. smegmatis* is capable of resisting phagocytosis until it becomes opsonized.

### 2.2. Opsonized M. smegmatis Induces the Release of Gelatinase Granules 

**Figure 2:**
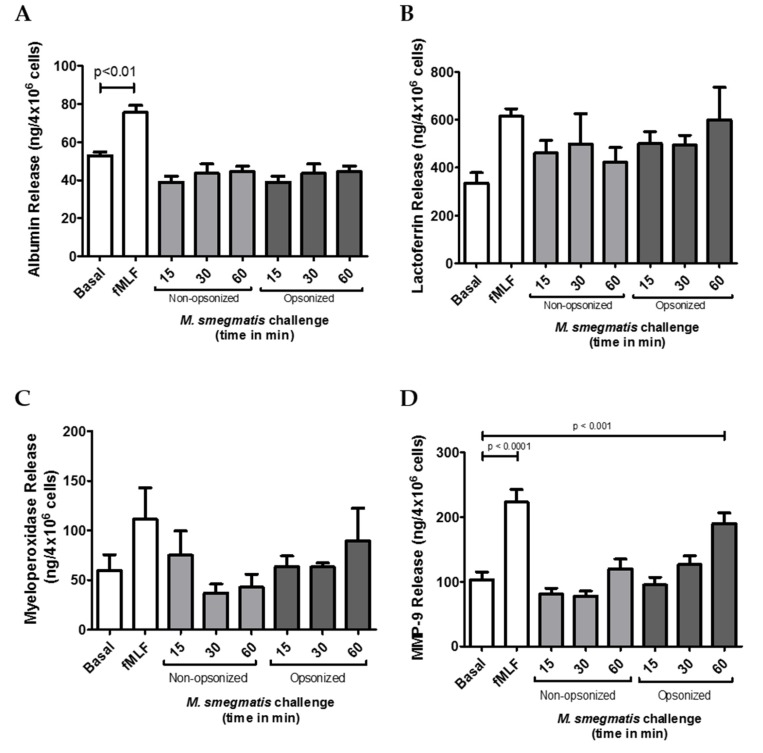
The effect of *M. smegmatis* challenge on granule exocytosis was tested by quantifying the content release of secretory vesicles (**A**), specific granules (**B**), azurophilic granules (**C**), and gelatinase granules (**D**) by ELISA. Quantities of granule content were measured in the supernatants from *M. smegmatis*-challenged neutrophils from four (A, B, C) and eleven (D) independent donors. Data are graphed as the mean ± SEM of release from 4 × 10^6^ human neutrophils.

Neutrophil granules are central contributors in the protection against bacteria, but if their release is not carefully controlled, degranulation can also lead to host tissue damage. Therefore, to evaluate the potential contribution of each granule to *M. smegmatis* pathology, the amount of content released from each granule subtype was tested in the supernatants of neutrophils challenged with *M. smegmatis*. Presence of albumin was quantified as a measure of secretory vesicle exocytosis (Figure 2A). Despite being the most easily mobilized neutrophilic granules, no secretory vesicle exocytosis was observed for either opsonized or non-opsonized *M. smegmatis* at any time point compared to basal levels. Similarly, exocytosis of specific granules, as measured by the release of lactoferrin (Figure 2B), and azurophilic granule exocytosis, as measured by myeloperoxidase release (Figure 2C), were also not significantly induced by either bacterial interaction at any time point. However, exposure of neutrophils to serum opsonized *M. smegmatis* elicited a time-dependent increase in MMP-9 release (Figure 2D). After 60 min of challenge with opsonized *M. smegmatis*, the concentration of extracellular MMP9 was significantly higher than basal conditions and reached similar levels of gelatinase release to the cells treated with fMLF, a known inducer of gelatinase degranulation.

### 2.3. M. smegmatis Does Not Cause or Inhibit the Release of Secretory Vesicles, Specific, or Azurophilic Granules at Early Time Points

**Figure 3:**
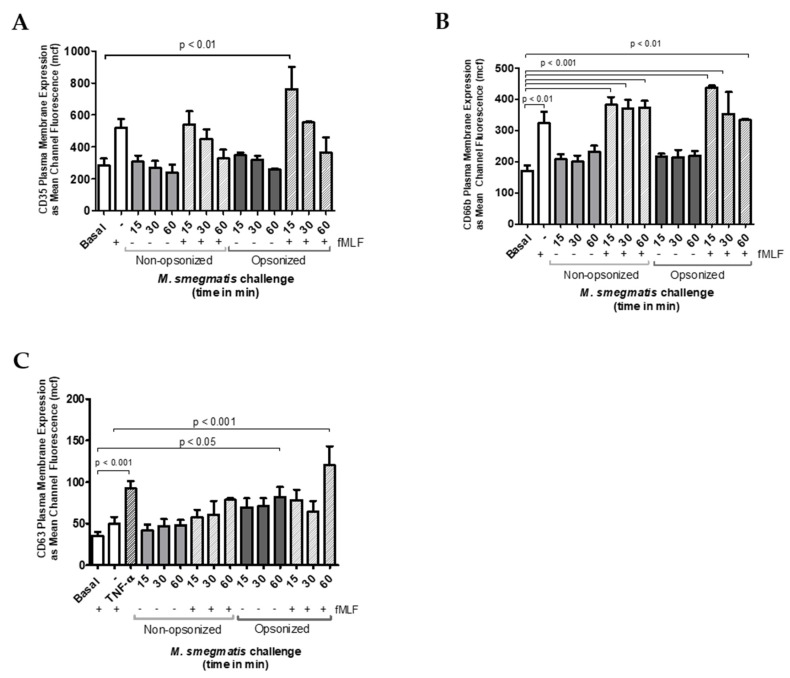
Neutrophils were unstimulated, stimulated with the bacterial peptide, N-Formylmethionine-leucyl-phenylalanine (fMLF), primed with TNFα followed by fMLF, or challenged with *M. smegmatis* at different time points (15, 30 or 60 min), or pre-treated with opsonized or non-opsonized *M. smegmatis*, for the indicated time points, followed by fMLF stimulation. Exocytosis of secretory vesicles (**A**), specific granules (**B**), and azurophilic granules (**C**) was measured via changes in surface membrane expression of CD35, CD66b, and CD63 markers, respectively, using flow cytometry. Data are expressed as the mean ± SEM of the mean channel fluorescence (mcf) for seven independent experiments for non-opsonized condition (A and B); for two independent experiments for opsonized condition (A and B); and for six independent experiments for C.

Exocytosis of secretory vesicles, specific and azurophilic granules was also confirmed using flow cytometry to measure the increase in membrane expression of specific markers for each granule type. Regardless of opsonization, co-culture of neutrophils with *M. smegmatis* did not increase the membrane expression of CD35, which is indicative of secretory vesicle exocytosis (Figure 3A), nor CD66b, which is exclusive to specific granules (Figure 3B). 

To test whether *M. smegmatis* was inhibiting the release of these granules, neutrophils were pre-treated with *M. smegmatis* for 15, 30, or 60 min, followed by stimulation with fMLF, a stimulus known to cause granule mobilization. Despite pre-treatment with *M. smegmatis*, CD35 and CD66b expression reached comparable levels of expression during fMLF stimulation alone, suggesting that the bacteria are not blocking the release of these granules. The time-dependent, decreasing trend in CD35 membrane expression in both bacterial treatment conditions is likely due to phagocytic events that remove available receptors for fluorescent antibody binding.

Changes in expression of CD63 were also measured as indicators of azurophilic granule exocytosis (Figure 3C). Non-opsonized *M. smegmatis* did not elicit an increase in expression independently, nor did it impair stimulation by fMLF. However, opsonized *M. smegmatis* increased expression of CD63 after 60 min, and enhanced expression even more when *M. smegmatis* co-culture was followed by fMLF stimulation. Regardless of the increase in membrane expression of this marker, azurophilic granule content was not found in the supernatants by ELISA (Figure 2C). 

### 2.4. Supernatants from M. smegmatis-Treated Neutrophils Degrade Extracellular Matrix Proteins

**Figure 4:**
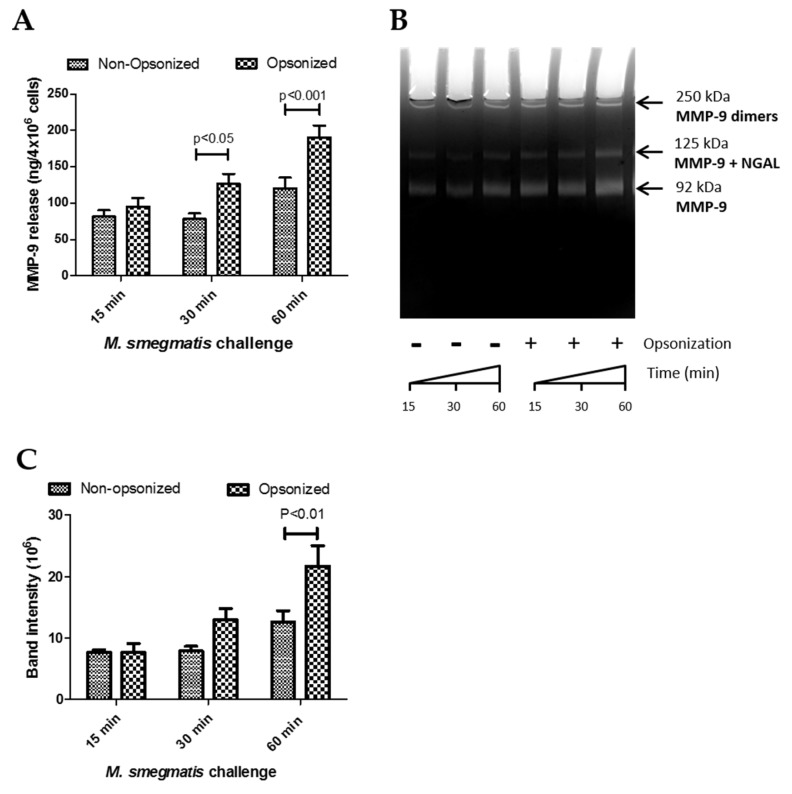
The effects of time and opsonization on Matrix Metalloproteinase 9 (MMP-9) release (data from Figure 2D) were re-analyzed by a two-way ANOVA with Bonferroni post-hoc analysis (**A**). Gelatin zymography of supernatants from neutrophils challenged with non-opsonized or opsonized *M. smegmatis* was performed on a 9% SDS-PAGE gel and stained with Coommassie Blue (**B**). The different isoforms of MMP-9 are indicated. Enzymatic activity was measured by quantifying the intensity of the clear bands where digestion took place (**C**). Data are graphed as the mean ± SEM of band intensity from four independent experiments. A two-way ANOVA of time and opsonization on MMP-9 release with Bonferroni post-hoc testing was conducted on this data.

Opsonized *M. smegmatis* caused significant release of MMP-9 compared to control conditions (Figure 2D). A two-way ANOVA was conducted on this data to examine the effect of time and opsonization on MMP-9 release (Figure 4A). Though there was no interaction effect between opsonization and time (*p* = 0.0873), simple main effect analysis showed that time (*p* < 0.0001) and opsonization (*p* < 0.0001) trends were both significant independently. Furthermore, Bonferroni post-hoc analysis further showed that opsonization caused significantly more release of MMP-9 compared to non-opsonized *M. smegmatis* at 30 and 60 min (Figure 4A). 

Next, to determine whether the higher induction of gelatinase granule exocytosis by opsonization of *M. smegmatis* resulted in the release of active MMP-9, the enzymatic activity of the supernatants collected for analysis by ELISA was assessed using gel zymography. The size of the band clearing corresponds to the degradative capacity of the supernatants from non-opsonized and opsonized-treated neutrophils (Figure 4B). Parallel to the ELISA data, exposure of neutrophils with opsonized *M. smegmatis* for 60 min resulted in the largest, most intense bands compared to lower time points with the opsonized bacteria and supernatants from neutrophils treated with non-opsonized *M. smegmatis*. The intensity of the bands was quantified by imaging software and analyzed by a two-way ANOVA to examine the effect of time and opsonization on band intensity as a measure of enzymatic activity (Figure 4C). There was no interaction effect (*p* = 0.6167), but the time (*p* = 0.0282) and opsonization (*p* = 0.0222) main effects were both significant. These data confirm the ELISA data observations and indicate that both opsonization and time affect gelatinase release, but that opsonization does not have the same effect at all values of time. 

Additionally, we confirmed that complement deposition, as opposed to other types of opsonization, was responsible for the effect on neutrophils. Gel zymography was performed with the supernatants from neutrophils exposed to either non-opsonized *M. smegmatis* or *M. smegmatis* opsonized with either complete human serum or heat-inactivated human serum (data not shown). Heat inactivation of the opsonizing serum resulted in similar levels of gel degradation as non-opsonized bacteria conditions, suggesting that complement deposition is the main reason for the change in neutrophil functional activity.

### 2.5. M. smegmatis Pre-Treatment Enhances Gelatinase Granule Exocytosis by Secondary Stimuli

**Figure 5:**
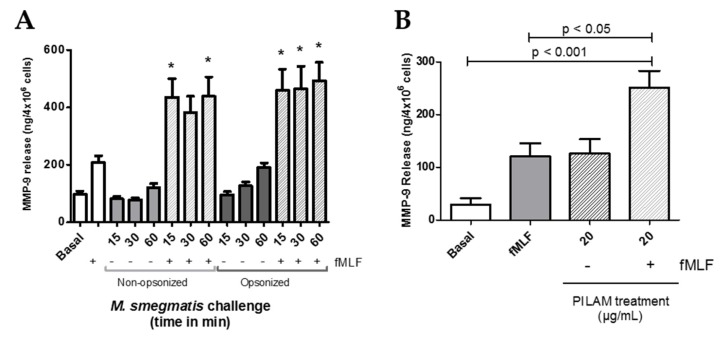
Gelatinase granule exocytosis was measured by content release of MMP-9 via ELISA after neutrophils were unstimulated (Basal); stimulated with fMLF, opsonized or non-opsonized *M. smegmatis*; or stimulated with fMLF in the presence or absence of opsonized or non-opsonized *M. smegmatis* at a MOI of 6:1 (**A**). MMP-9 release was also measured in the supernatants from neutrophils exposed to PILAM for 30 min, or fMLF after PILAM pre-treatment (**B**). Data are expressed as the mean ± SEM of ng/mL of gelatinase release; * denotes *p* < 0.05 as compared to fMLF stimulation alone.

Formylated peptides, like fMLF, are derived from bacterial protein degradation and can act as potent neutrophil chemoattractants and activators. Furthermore, the intensity of neutrophil responses to fMLF stimulation can be modulated by exposure to other activating stimuli. To assess if *M. smegmatis* exposure can affect the release of gelatinase by a secondary stimulating agent, neutrophils were pre-treated for a time course with opsonized and non-opsonized *M. smegmatis* followed by challenge with fMLF (Figure 5A). *M. smegmatis* treatment does not cause complete release of gelatinase content independently, but regardless of bacterial opsonization, exposure to the bacteria enhances the release of MMP-9 to secondary fMLF stimulation. Notably, despite a smaller population of neutrophils interacting with non-opsonized *M. smegmatis*, upon fMLF stimulation, the gelatinase release was comparable to neutrophils that were pre-treated by opsonized bacteria.

### 2.6. M. smegmatis Cell Wall Glycolipid Stimulation Causes Gelatinase Granule Release

Mycobacteria are known to shed their cell wall glycolipids and modulate immune system functions. Therefore, to define *M. smegmatis* factors that contribute to observed neutrophil functional responses, we tested the exocytosis of each granule after neutrophil exposure to a *M. smegmatis* surface glycolipid, phosphoinositol lipoarabinomannan (PILAM). Like the whole bacteria, treatment of neutrophils to PILAM did not cause the exocytosis of secretory vesicles nor specific and azurophilic granules (data not shown). However, addition of 20 ug/mL of PILAM caused the selective degranulation of only gelatinase granules, which reached the level of the positive control, fMLF (Figure 5B). Also, the mycobacterial glycoplipid’s immunomodulatory properties on gelatinase granule exocytosis were tested by pre-treating neutrophils with PILAM for 30 min, followed by stimulation with fMLF (Figure 5B). fMLF-mediated release of MMP-9 was enhanced by pre-treatment with PILAM, but this could represent an additive effect by both stimuli. Other cell wall components and complement recognition are likely contributing to the neutrophil responses observed; but recognition of PILAM plays a direct role in the release of gelatinase granules on exposure to *M. smegmatis* in vitro.

### 2.7. M. smegmatis Induces Gelatinase and Specific Granule Exocytosis after Extended Timepoints

**Figure 6:**
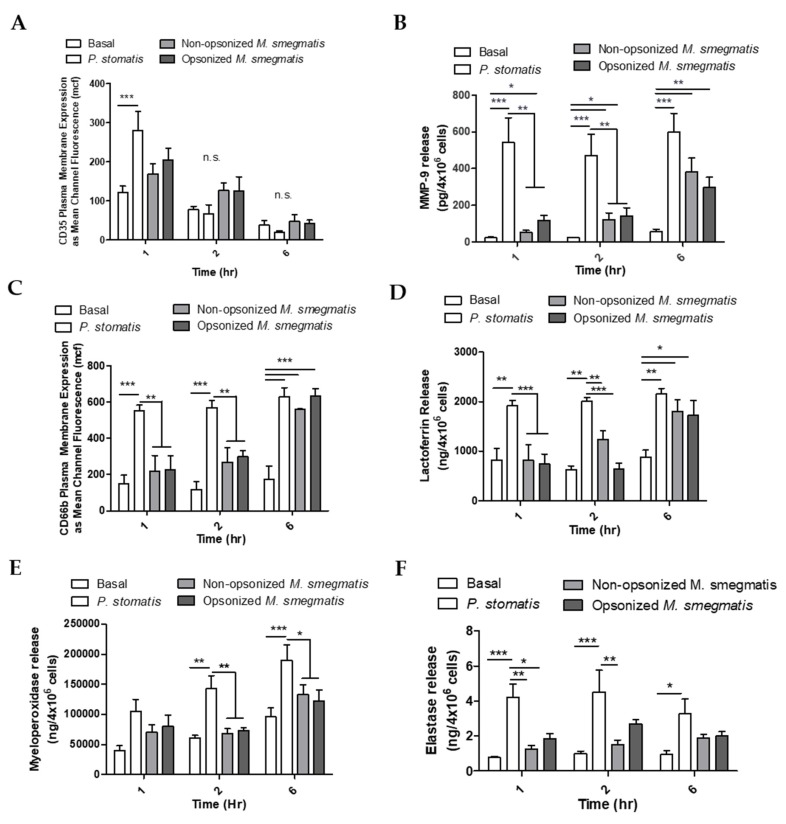
Neutrophils were unstimulated (basal), stimulated with *Peptoanaerobacter stomatis* (MOI 10), or different treatments of *M. smegmatis* (MOI 6) for 1, 2, or 6 h. Exocytosis of secretory vesicles was measured by flow cytometry as the surface membrane expression of CD35 (**A**). Gelatinase granule exocytosis was measured as the presence of MMP-9 in the supernatants of neutrophils by ELISA (**B**). Specific granule exocytosis was measured by flow cytometry as the surface membrane expression of CD66b (**C**) and the presence of Lactoferrin in the neutrophil supernatants by ELISA (**D**). Azurophilic granule release was measured as the presence of Myeloperoxidase (**E**) and Elastase (**F**) in the supernatants of neutrophils. The histograms in A and C show the flow cytometry data as the average mean channel fluorescence (mcf) ± SEM of three independent experiments. B, D, E, and F show the average release of each granule marker ± SEM from five independent experiments. * = *p* value < 0.05, ** = *p* value < 0.01, *** = *p* value < 0.001.

To determine whether the pattern of neutrophil degranulation by *M. smegmatis* differed at longer timepoints, granule exocytosis was measured by flow cytometry and ELISA in neutrophils cultured with non-opsonized or opsonized *M. smegmatis* for 1, 2, and 6 h. As a positive control, neutrophils were cultured with *Peptoanaerobacter stomatis*, a putative oral pathogen that has been shown to induce robust exocytosis of all four neutrophil granules [32]. In the case of secretory vesicles, there was a time-dependent decrease in the expression of the secretory vesicle marker on the membrane of neutrophils in all conditions tested (Figure 6A). This decrease in CD35 membrane expression could be due to shedding of the receptor as the cell ages, or phagocytic events that remove available receptors for fluorescent antibody binding. Neutrophils challenged with *M. smegmatis* did not induce significant release of secretory granules at any point in the time course tested. 

As a measure of gelatinase granule exocytosis, we measured the levels of MMP9 in supernatants of neutrophils cultured with media, *P. stomatis*, or *M. smegmatis* by ELISA (Figure 6B). *P. stomatis* induced a robust release of MMP9 from neutrophils. At one hour, opsonized *M. smegmatis* caused a larger release of MMP9 than non-opsonized bacteria. This difference between non-opsonized and opsonized *M. smegmatis* decreased in magnitude as the time course progressed, leading to significant gelatinase granule exocytosis by six hours. While *P. stomatis* challenge resulted in specific granule exocytosis at 1, 2, and 6 h, neutrophils challenged with *M. smegmatis* did now show exocytosis until 6 h as tested by flow cytometry (Figure 6C) and ELISA (Figure 6D). Azurophilic granule exocytosis was measured by ELISA as myeloperoxidase (Figure 6E) and elastase (Figure 6F) activity in the supernatants. Challenge with *P. stomatis* induced the release of both myeloperoxidase and elastase activity, but *M. smegmatis* did not evoke these granules at any timepoint tested. 

## 3. Discussion

*M. smegmatis* only rarely causes severe infections; however, these infections are difficult to treat due to misdiagnosis and intrinsic mycobacterial cell wall-mediated resistance to antibiotic regiments. In reported cases of *M. smegmatis* infection, histopathological analysis shows skin ulceration and subcutaneous necrosis, with treatment involving aggressive surgical debridement of the infected tissue [12]. Neutrophils are recognized as initial effectors of acute inflammation during mycobacterial infections [33,34,35,36,37]. For example, neutrophils were recruited as early as 15 min after *M. smegmatis* subcutaneous inoculation in a BALB/c mouse model [38]. In vitro, neutrophils exposed to *M. smegmatis* ATCC 19420 released pro-inflammatory cytokines including TNF-α, IL-6, and IL-8 [28] that act as chemotactic signals to recruit other neutrophils. Thus, as with other bacteria, neutrophils likely play a central role in the host defense against *M. smegmatis*. We therefore began by characterizing neutrophil granule release evoked by *M. smegmatis.* We found that opsonized bacteria caused the extracellular mobilization of gelatinase containing granules at early time points, while specific granules were mobilized at later time points. These activities potentially contribute to the characteristic tissue pathology in these rare infections. 

After their rapid accumulation at sites of bacterial infection, neutrophils bind and ingest microbes. Our results indicate that serum opsonization is important, and that human neutrophils do not efficiently phagocytize non-opsonized *M. smegmatis* in vitro. This could reflect a survival mechanism used in soil and water environments where these bacteria normally reside. In water, free-living amoeba feed on bacteria by phagocytosis, but many environmental mycobacteria resist this predation by blocking uptake by protists [39,40]. Opsonization with human serum prior to neutrophils interaction appears to circumvent these strategies. Our results are consistent with a previous report of *M. smegmatis* ATCC 607-neutrophil interactions that showed that after 30 min of co-culture, at a multiplicity of infection of 50 bacteria per neutrophil, only 25% of neutrophils phagocytized non-opsonized *M. smegmatis.* With opsonization, 60% of phagocytes showed intracellular bacteria [26]. Here we expand these observations and confirm that serum opsonization is necessary for efficient phagocytosis by human neutrophils. 

Previous publications have focused on the microbicidal capacity of the granule matrix content and characterized granule trafficking to the *M. smegmatis*-containing phagosome [25,26]. However, *M. smegmatis*-triggered granule exocytosis had not previously been directly assessed. Measurements of the granule content in the supernatant of *M. smegmatis*-stimulated human neutrophils and changes in granule-specific plasma membrane markers indicated that only interactions with opsonized *M. smegmatis* produced significant exocytosis of gelatinase granules at early timepoints, a response previously demonstrated with purified mycobacterial PILAM. The exocytosis of gelatinase granules is critical to neutrophil extravasation from blood vessels into tissue partly due to the release of collagenase and matrix metalloproteases that allow phagocytes to penetrate the basement membrane of the endothelium [23]. Previous reports show that during neutrophil co-culture with *M. smegmatis*, 60%–80% of bacteria are eliminated within an hour, which is comparable to neutrophil killing of other Mycobacteria and *Staphylococcus aureus* [29,41,42,43]. Coupled with our results from this study, this suggests that even though neutrophils can efficiently kill internalized opsonized *M. smegmatis,* there is also significant release of active matrix metalloproteinases. Nonetheless, further studies are needed to determine whether this contributes to the tissue degradation and abscess formation seen in *M. smegmatis* infections. Tissue damage by uncontrolled or prolonged gelatinase granule exocytosis has already been implicated with the progression of pathology in asthma, rosacea, rheumatoid arthritis, and periodontal diseases [23,44,45]. When the exocytosis of neutrophil granules was followed over a longer time course, MMP9 from gelatinase granules was detected in the supernatants at all timepoints. In contrast neutrophils specific granule content was not detected until 2 to 6 h after *M. smegmatis* exposure. The azurophilic granule membrane marker CD63 was detected by flow cytometry at 1 h after opsonized *M. smegmatis* challenge, but no significant release of the two granule matrix proteins MPO and elastase was detected at 1–2 or 6 hours post-bacterial challenge. We conclude that *M. smegmatis*, independent of opsonization or time, does not induce azurophilic granule exocytosis in human neutrophils. 

Neutrophil granule exocytosis is tightly controlled, where the release of the different granule subtypes is based on stimulus intensity. Secretory vesicles are the easiest to evoke and show the most complete exocytosis, while azurophilic granules are the most tightly regulated with limited exocytosis because they contain the most toxic components [46]. Therefore, it was surprising that neither PILAM nor *M. smegmatis* induced the release of secretory vesicles. The exocytosis of secretory vesicles enriches the neutrophil plasma membrane with different types of immune receptors that are needed to help the cell to extravasate from blood vessels into surrounding tissues and enhance the sensitivity of neutrophils to different stimuli [47]. Immune cells recognize mycobacteria like *Mycobacterium tuberculosis* in part through Toll-like receptors (TLR), Nod-like receptors (NLR), and C-type lectin receptors, while Fc receptors and complement receptors recognize opsonized tubercle bacilli [48], and many of these receptors are present in secretory vesicles [47]. Nonetheless, it is possible that the number of receptors present at basal levels is sufficient to recognize *M. smegmatis* or PILAM and induce other neutrophil functional responses. 

To the best of our knowledge, the immediate pattern of exocytosis of human neutrophil granules is unique to *M. smegmatis*. While other bacteria have been shown to block or induce degranulation of some or all neutrophil granule subtypes [32,49,50,51,52,53,54,55,56,57,58,59], release of only gelatinase granules at early time points has not been seen with bacteria other than *M. smegmatis*. An important distinction is that *M. smegmatis* is not preventing granule release to other stimuli, since granule mobilization was not affected upon fMLF stimulation by human neutrophils cultured with *M. smegmatis*. Thus, it will be important to determine which signaling pathways are activated by *M. smegmatis* challenge, and how they can specifically induce gelatinase granule exocytosis at early time points. Increases in intracellular calcium and actin reorganization, for example, are required for the release of all granule subtypes. The dependence of exocytosis on granule-specific signaling mechanisms is currently an area that is not yet well understood [60,61]. 

Previous studies on the interactions between *M. smegmatis* and human neutrophils have focused on the intracellular killing of phagocytized bacteria. Our study is unique in that it examines the neutrophil granule exocytosis response that potentially also damages host tissues. Our results provide evidence that live *M. smegmatis* produces an unprecedented pattern of human neutrophil degranulation by exclusively inducing gelatinase granule release during an initial interaction. Interestingly, further interaction between neutrophils and *M. smegmatis* induced specific granule exocytosis. As such, we speculate that the neutrophil exocytosis of matrix metalloproteinases including gelatinase contributes to the pathology of human soft tissues infections. If so, efforts to target this activity may be valuable in improving outcomes in these rare infections. 

## 4. Materials and Methods 

*Human neutrophil isolation:* Human neutrophils were isolated from healthy donors using Plasma-Percoll gradients as previously described [62], and used within 3 h. Trypan blue exclusion indicated >97% viability for isolated cell populations, which contained >95% neutrophils as judged by microscopic examination. Human donor recruitment, blood draws and the use of the material were performed according to guidelines reviewed and approved by the Institutional Review Board of the University of Louisville.

*Mycobacterial culture: M. smegmatis* strain mc^2^ (gift from Bill Jacobs) was grown to mid-logarithmic phase in 5 mL of Middlebrook 7H9 ADC medium with 0.05% Tween-80 (ADC is 50 g/mL bovine serum albumin fraction V (BSA), 75 g/mL D-glucose, and 8 g/mL NaCl) at 37 °C in 16 mL glass tube on a tube roller with a loose cap for 24 h. The roller tube with the *M. smegmatis* culture was allowed to sit on the benchtop for 20 min, and only the top 3 mL of the culture were extracted to be used for the neutrophil inoculum. This 3 mL was further treated by passing the culture through a 21 gauge syringe a couple times to remove most bacterial aggregates prior to dilution, as verified by confocal microscopy.

Bacterial concentrations were estimated by measuring optical density (OD at A600) using a previously established formula of bacteria/mL/OD. *M. smegmatis* was added to suspended neutrophil cultures at a multiplicity of infection (MOI) of 6:1. To visualize bacteria by confocal microscopy, multi-copy plasmid pMN437 expressing gfp2+ [63] from the Psmyc promoter was electroporated into *M. smegmatis* mc2. A transformant was isolated and grown in the presence of 50 μg/mL hygromycin and characterized for stable fluorescence. 

*Opsonization of M. smegmatis inoculum*: Bacteria were again centrifuged at 6000 g for 5 min at room temperature, and pellets resuspended in a 1:1 mix of pooled human serum (Complement Technology NHS, Tyler, TX, USA) and sterile PBS. Bacteria were incubated without agitation for 20 min at room temperature with the serum mixture, and then washed twice with PBS. 

*Phagocytosis of mycobacteria:* Neutrophils (cells/mL) were challenged with non-opsonized or opsonized GFP-expressing *M. smegmatis* (MOI 6) in Krebs+ buffer (120 mM NaCl, 4.8 mM KCl, 5.5 mM dextrose, 3.12 mM monobasic NaPO_4_, 12.48 mM dibasic NaPO_4_, 0.54 mM CaCl_2_ and 1.2 mM MgSO_4_ dissolved in deionized water at pH 7.4 and kept at 4 °C in depyrogenated bottles) for 60 min in a shaking water bath at 37 °C. After the incubation, each sample was transferred on top of a heat-inactivated, human serum-coated coverslip inside the well of a 24-well plate. To attach the cells to the coverslip, the plate was centrifuged for 4 min at 600 g with maximum breaking in a refrigerated tissue culture centrifuge cooled to 14 °C. The remaining liquid was aspirated, and the coverslips were incubated with 5 µg/mL of Alexa Flour 647-conjugated Wheat Germ Agglutinin (WGA, Molecular probes W32466) for 10 min in the dark. Excess WGA was removed by washing the coverslips twice with PBS. The attached cells were fixed using 10% formalin for 10 min followed by one wash with PBS. Coverslips were attached to slides using an anti-fade adhesive and imaged using confocal microscopy where Z plane stacking was used to confirm the intracellular location of bacteria. Specifically, cells were counted from different fields and classified by whether they had internalized, attached, or no associated bacteria. The number of internalized and attached bacteria was also counted. 

For phagocytosis efficiency analysis on the ImagestreamX (Amnis, Seattle, WA), neutrophils were challenged with GFP-expressing *M. smegmatis* (MOI of 6) like above for 15, 30 or 60 min, or with different MOI (6, 50, 100) for 60 min. After co-culture, cells were then washed with FTA buffer (BD 211248, Franklin Lakes, NJ, USA) with 0.05% sodium azide followed by fixation with 1% formalin. On the imagestreamX, GFP fluorescence was excited at 488 nm and recorded on the 480–560 nm channel (CH2) as previously described [64]. A total of 2000 events were collected from a gate for the neutrophil population based on size. Further analysis was done using the IDEAS software (Amnis) to only measure GFP signal intensity coincident with the inside of the neutrophil cell membrane as a measure of internalized bacteria using the internalization wizard. 

*Neutrophil granule exocytosis:* The supernatants of unstimulated, fMLF-stimulated (300 nM, 5 min), *Peptoanaerobacter stomatis*-stimulated (MOI 10) or *M. smegmatis*-stimulated (MOI 6) neutrophils (4 × 10^6^ cells/mL) were collected, mixed with 1% phosphatase and 1% protease inhibitors, and stored in sterile microcentrifuge tubes at −80 °C. Treatment with the formylated bacterial peptide, fMLF, or the oral bacteria *P. stomatis* was used as a positive control. These supernatants were tested for degranulation by using commercially available enzyme-linked immunosorbent assays (ELISAs) to measure the amount of albumin (secretory vesicles, Abcam 108788), MMP-9 (gelatinase granules, Boster EK0465), lactoferrin (specific granules, Abcam 108882), myeloperoxidase (azurophilic granules, Abcam 119605), and elastase (Azurophilic granules, Abcam 119553).

The exocytosis of azurophil granules, specific granules, and secretory vesicles was also determined by measuring the increase in the presence of fluorescently tagged granule markers (CD63, CD66b, and CD35 respectively) on the cell plasma membrane with a FACSCalibur flow cytometer as previously described [65,66]. Treatment with fMLF or TNF-α + fMLF were used as positive controls. Neutrophils’ (4 × 10^6^ cells/mL), unstimulated, stimulated with fMLF (300 nM, 5 min, Sigma, St. Louis, MO, USA) or *P. stomatis* (MOI 10), challenged with *M. smegmatis* for 15, 30, 60 min, 2 h or 6 h or pretreated with *M. smegmatis* for 15, 30, or 60 min followed by stimulation with fMLF. After stimulation, cells were incubated on ice in the dark for 45 min with fluorochrome-conjugated antibodies specific to each granule marker. These were fluoresecein isothiocyanate (FITC)-conjugated anti-CD63 (azurophil granule, Ancell 215-040, Stillwater, MN, USA), FITC-conjugated anti-CD66b (specific granule, Biolegend 305104, San Diego, CA, USA), and phycoerythrin (PE)-conjugated anti-CD35 (secretory vesicle, Biolegend 333406, San Diego, CA, USA). Following antibody incubation, cells were washed with 0.5% sodium azide (S2002, Sigma, St. Louis, MO, USA) in FTA buffer (211248 BD, Franklin Lakes, NJ, USA) and fixed with 1% paraformaldehyde (PX0055-3, EMD, Darmstadt, Germany). 

*Phosphoinositol lipoarabinomannan (PILAM):* PILAM was purchased from Invivogen (#tlrl-lams, San Diego, CA, USA) in powder form. The PILAM powder was dissolved in sterile endotoxin-free deionized water to a concentration of 1 mg/mL and stored at −20 °C. For all experiments involving PILAM, neutrophils were exposed to 20 μg/mL for 30 min. 

*Gel zymography:* The enzymatic activity of the supernatants of non-opsonized or opsonized *M. smegmatis*-stimulated (MOI 6) neutrophils (4 × 106 cells/mL) was tested by gel zymography using a 9% SDS-PAGE gel saturated with 1 mg/mL gelatin (Sigma, G9391) as previously described [67]. Equal volumes from each condition were loaded onto the gel and electrophoresed on ice for 30 min in 1X SDS-PAGE gel running buffer at a constant 50 V followed by 2.5 h at 100 volts. The gel was incubated in 2.5% Triton X-100 in dH2O for an hour at room temperature followed by an overnight incubation at 37°C in substrate buffer (50 mM Tris pH8.8, 10 mM CaCl_2_). The gel was stained in 0.5% Coomassie Blue R-250 in water/methanol/acetic acid (45:45:10) for 30 min and destained with 50% methanol in dH2O. The gel was rehydrated with dH2O and imaged on a ChemiDoc XRS+ (Bio-Rad Hercules, CA, USA). The captured image was converted to black and white for best visibility of the bands. Densitometry measurements were performed using the Image Lab software from the ChemiDoc. 

*Statistical analysis:* Statistical differences among experimental conditions and time points were analyzed by a one-way ANOVA and the post-hoc Tukey multiple-comparison unless otherwise noted. In those cases, a two-way ANOVA and Bonferroni post-hoc testing was applied using GraphPad Prism Software (Graphpad San Diego, CA, USA). Differences were considered significant at the level *p* < 0.05.

## Figures and Tables

**Table 1:** Internalization of *Mycobacterium smegmatis* by human neutrophils.

	None Associated	Internalized	Attached	Internalized and Attached	Total Neutrophil Counts
Non-opsonized	220 (69%)	46 (14%)	29 (9%)	26 (8%)	321
Opsonized	58 (19%)	121 (40%)	11 (4%)	115 (38%)	305

Note. Neutrophils were counted from two experiments and classified by the location of their associated bacteria.
